# Anatomy of vertical heteroepitaxial interfaces reveals the memristive mechanism in Nb_2_O_5_-NaNbO_3_ thin films

**DOI:** 10.1038/srep09229

**Published:** 2015-03-18

**Authors:** Linglong Li, Lu Lu, Zhiguang Wang, Yanxi Li, Yonggang Yao, Dawei Zhang, Guang Yang, Jianjun Yao, Dwight Viehland, Yaodong Yang

**Affiliations:** 1 Multi-disciplinary Materials Research Center, Frontier Institute of Science and Technology, Xi'an Jiaotong University, Xi'an 710049, China; 2 Electronic Materials Research Laboratory, Key Laboratory of the Ministry of Education & International Center for Dielectric Research, Xi'an Jiaotong University, Xi'an, 710049, China; 3 Department of Materials Science and Engineering, Virginia Tech, Blacksburg, Virginia, 24061, USA; 4 Asylum Research, Oxford Instruments Company, Shanghai 200233, China

## Abstract

Dynamic oxygen vacancies play a significant role in memristive switching materials and memristors can be realized via well controlled doping. Based on this idea we deposite Nb_2_O_5_-NaNbO_3_ nanocomposite thin films on SrRuO_3_-buffered LaAlO_3_ substrates. Through the spontaneous phase separation and self-assembly growth, two phases form clear vertical heteroepitaxial nanostructures. The interfaces between niobium oxide and sodium niobate full of ion vacancies form the conductive channels. Alternative I-V behavior attributed to dynamic ion migration reveals the memristive switching mechanism under the external bias. We believe that this phenomenon has a great potential in future device applications.

As the fourth fundamental passive circuit element, memristors and memristive devices were initially predicted by Chua in the early 1970s^1^,^2^, but were not realized until 2008 when the bipolar-resistive switching phenomenon was found in TiO_2-x_^3^. Similar concepts such as bistable switching and voltage-controlled resistance phenomenon were also reported in a variety of oxides such as ZnO^4^, Al_2_O_3_^5^, TiO_2_^6^, Nb_2_O_5_^7^,^8^, and tantalum oxide^9^,^10^. Furthermore, this multistate switching phenomenon was also found in perovskite and chemical modified polymer, namely various tunable modes of the electronic conducting states^11^,^12^,^13^,^14^. But few works refer to a ferroelectric- semiconductor composite whose mutual coupling effect under electric fields may bring interesting I–V characteristics. The main feature of ferroelectrics is the spontaneous polarization which can be governed by an external electric field and has a memory even after the removal of the field. While the most significant character of semiconductor oxide is the electric-field-excited carriers. Combining these two together may bring new functionality under external electric field. Polarization induced by external electric field will form an internal electric field that can affect semiconductor phase. This intermediate electric coupling interaction in the composite, similar to the strain-media in a magnetoelectric composite material^15^, may produce novel electric properties, e.g., memristor.

How to obtain an elaborate arrangement of these two different phases pursuing for optimal functionality becomes a thorny question. Composite thin films usually share three different configurations: 2-2 laminate composite, 0–3 particulate composite and 1–3 fiber/rod composite^15^. Vertical nanocomposite heteroepitaxial 1–3 structure is a promising way to enhance the interaction between two phases, because it has much larger interfacial area and suffers less substrate clamping effect compared to conventional multilayer 2-2 structures. Vertical nanopillar microstructure was firstly demonstrated in the La_0.7_Ca_0.3_MnO_3_ (LCMO) and MgO system^16^. Originated from the second phase of MgO, tensile stress tuned the magnetotransport properties of manganite nanoclusters. Another 1–3 type composite, magnetostrictive CoFe_2_O_4_ nanopillars randomly embedded in a piezoelectric BaTiO_3_ matrix was synthesized too^17^. Then self-assembled composite materials become popular in perovskite ferroelectric based multifunctional materials research area. To fabricate nanocomposite thin films with strong coupling effect in self-assembly forms, we need to select suitable materials, substrates, and control their microstructure and interface accurately. Evidence shows that sharing with similar or multiple relationships in lattice parameter and crystal orientation, different phases can lead to a stable crystal structure during phase separation growth^18^.

In this work, to design a 1–3 type self-assembly composite thin films that can response to the external electric field, we select sodium niobate (NaNbO_3_) as the ferroelectric matrix. NaNbO_3_ gains its popularity due to its high Curie temperature being regarded as a desirable property for lead-free piezoelectric materials^19^,^20^,^21^,^22^. On the other hand, niobium oxide already fulfills its potential as a new kind of metallic oxide memristor^3^,^23^. Thus, combination of these two significant materials would open a vitally new horizon to develop new memristor devices. Another inspiring stimuli is that Wang et al. reported that NaNbO_3_-Nb_2_O_5_ (NNO-NO) can grow epitaxially in thin films^24^, and Yan et al. reported that these two can be composited together in nanotubes^25^. But in these previous works, due to complex phases distributions, their growth mechanism, interaction between NNO, NO and substrate are still not clear. Importantly, the local electrical performance of this NNO-NO nanocomposite is lacking, thus there is no clue to discover new electric properties. After depositing the NNO-NO thin film via pulsed laser deposition, atomic force microscopy (AFM) images provide direct evidence to confirm the two phases separation from a top view. The interface and interaction between two phases and substrate are denoted by high angle annular dark field (HAADF) images.

## Results

**Figure 1:**
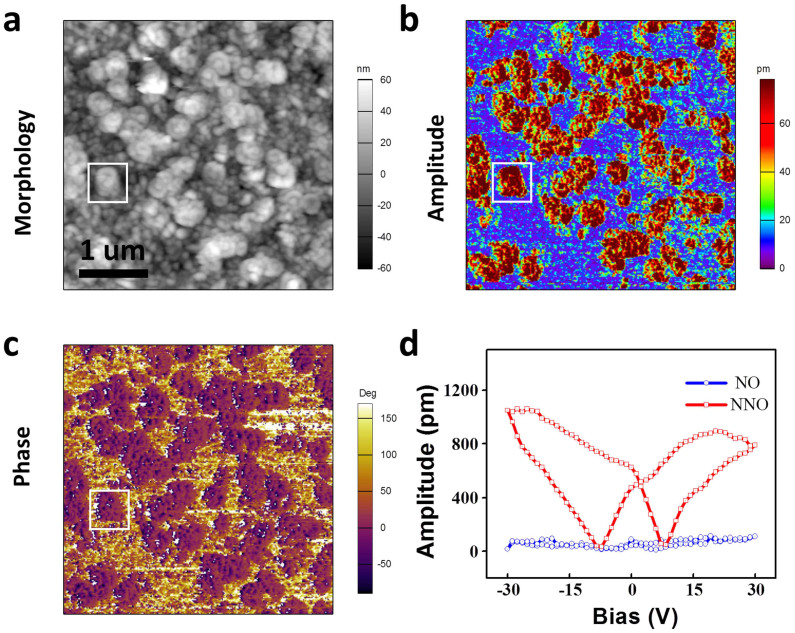
Ferroelectric properties of the NNO-NO thin film: (a) top-view morphology scanned by PFM; corresponding piezoresponse amplitude signal mapping (b) and piezoresponse phase signal mapping (c). (d) piezoresponse amplitude versus bias curves of NNO and NO regions.

An important starting point for our studies is to determine if this composite material has the ability to form heteroepitaxial nanocomposite. Phase separation during self-assembly growth is probed by piezoresponse force microscope (PFM) images from top view (as shown in figure 1a–c). There are two different morphologies in figure 1a. Some hundreds of nanometers, round shape grains (selected by a white square) distributed in a matrix randomly. The matrix consists of small grains with diameter of dozens of nanometers. From the piezoresponse amplitude image (figure 1b), we can infer that these round grains with higher piezoresponse (dark red color in figure 1b) are ferroelectric NNO phase, and matrix with very low piezoresponse signal (blue color in figure 1b) is NO phase. Similarly, two different contrasts in figure 1c suggest the phase separation. Comparison details of piezoresponse amplitude versus bias curves from different NNO and NO regions are provided in figure 1d. It's obvious that red curve (signal comes from red region in figure 1b) has a strong piezoresponse, while blue curve (signal comes from blue region in figure 1b) is much weaker.

**Figure 2:**
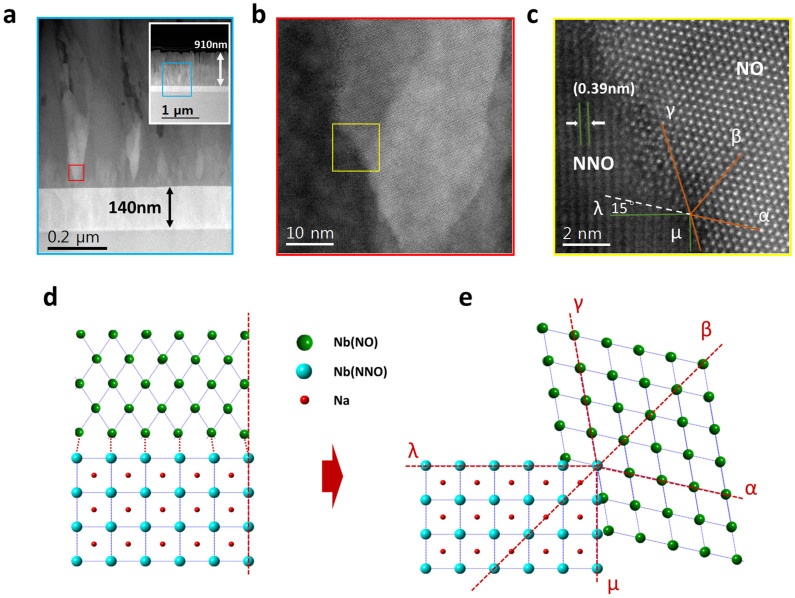
Inset in (a) is a cross-sectional image of the SRO buffered NNO-NO thin film on LAO substrate, (a) and (b) are higher magnification HAADF-STEM images of this area.(c) High resolution Cs-corrected STEM image of a NNO and NO boundary. Illustrations of different growth modes: (d) ordinary bottom-top growth and (e) rotated growth minimizes the mismatch symmetrically, proposed base on observation in (c).

Phase distributions and microstructures inside the thin film are probed further by transmission electron microscopy and a cross-section STEM sample was prepared by FIB. Figure 2a and inset show the 910 nm thick thin film with a 140 nm SrRuO_3_ buffer layer as the bottom electrode. There are some cone-like bright regions surrounded by some dark ones. After increasing magnification in this region (figure 2b and 2c), well-defined contrast of two separated phases can be observed distinctly. In fact, the crystal structures of pseudocubic NNO (lattice parameters: a = 3.91 Å) and orthorhombic NO phase (lattice parameters of a, b, c are 6.618 Å, 29.312 Å, 3.936 Å, respectively) are quite different, it is difficult to achieve epitaxial growth. In an ordinary epitaxial growth, top and bottom layers usually share the same axis, which is perpendicular to the bottom substrate (figure 2d). If so, the 20% mismatch between NNO and NO is too large to keep a good growth from collapsing. A distinct style that we grow NNO and NO together and remain their epitaxial relationship is adopted to minimize the mismatch and obey the law of crystallization kinetics. Figure 2c shows the details of the interface between two different phases. The brown lines denoted as α, β and γ mark the crystallographic planes of the NO phase, and the interplanar spacing are 0.319 nm, 0.319 nm, 0.304 nm, belonging to {180}, {180}, {200} respectively. The included angle between α and β is 61.13°, and 59.43° for that between β and γ directions. The green lines marked as μ and λ show the family of {100} planes of NNO phase. The relationship and interaction between NNO and NO phases can be considered in this way: β direction in NO phase, actually is the [110] direction in NNO phase. It is the shared axis of the epitaxial growth for both two phases symmetrically. Growing along this diagonal direction, composite thin film can minimize and keep same angles between γ and μ, α and λ as 15°. To make a better anatomy of the growth mechanism, we draw a crystal structure illustration (figure 2e) and set up the same coordinate drawing from figure 2c. In figure 2d two layers share a common axis perpendicular to the substrate (the red dash line), but in figure 2e, two phases have a normal rotated 45° from the substrate (line β). It decreases the mismatch between these two phases from 20% to only 11.6% (along β direction).

**Figure 3:**
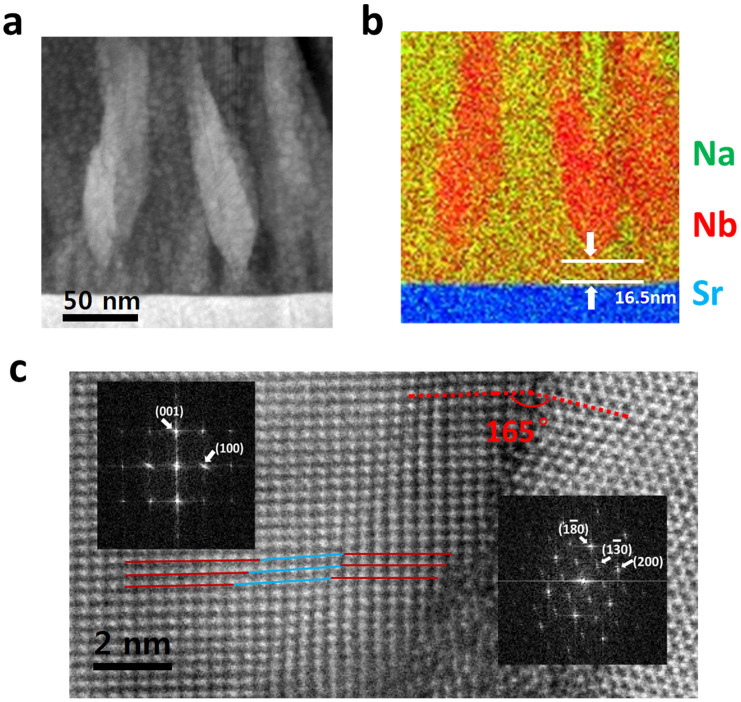
(a) STEM image of cone-like structures and (b) EDS element mapping of the same area. Red color: Nb, Green: Na, Blue: Sr. (c) HR-STEM image of NNO and NO phases boundary. Insets are their FFT patterns from different zones.

Figuring out that the mismatch reduction in growth is attributed to the 15° boundary formed by NNO and NO phase, we want to gain in-depth view about their relationship with the bottom layer. From the EDX mapping, we can know that the strong niobium signal (represent the niobium oxide beneficiation) appears at 16.5 nm above the bottom layer (figure 3b). Clearly, NO does not disperse well on SRO, and the surface of SRO is almost covered by NNO at the beginning of deposition process. In fact, the different wetting abilities of these two phases give a big favor to reduce the mismatch between NO and SRO in crystallization kinetics. Perovskite NNO phase has lattice parameter and structure similar to the buffer layer which offers a better wetting property than NO phase at the beginning. So NO grows from NNO rather than SRO, consequently NNO and NO form a suitable interface to allow both of them grow simultaneously. Figure 3c shows the critical boundary between NNO and NO phases. These two phases form (red broken line) a 15° angle to connect with each other, same as the angle between direction α and λ in figure 2c. Even so, the lattices in NNO still have some distortion in order to further release the inherent strain (solid blue lines in figure 3c). Fast Fourier transform (FFT) images of right part (NO phase) and left part (NNO phase) with several representative spots are shown in insets. The FFT pattern of NNO phase also shows some distortion at (100). Another interesting finding is that at the beginning of deposition process the majority of the thin film is NNO phase (as shown in figure 3b), but from the top view, we can figure out that NO phase takes place of NNO and becomes the majority or matrix (PFM image in figure 1b). This phenomenon can also be attributed to the different wetting abilities of NNO and NO compared with the SRO layer, resulting in the final cone-like distribution of NO phase in the NNO matrix.

**Figure 4:**
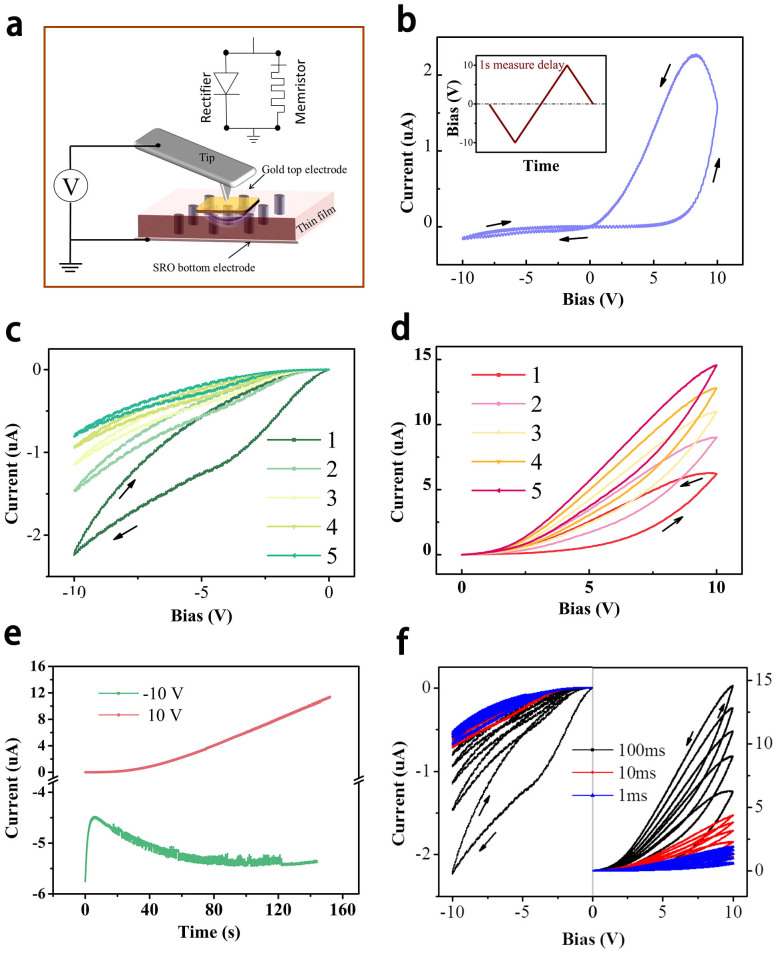
Electrical properties of the NO and NNO composite thin film to show the memristive switching process: (a) Schematic of the junction with the electrodes and tip. The feasible theoretical model is also provided as an inset. (b) The initial I–V curve of the device. Initiative 5 I–V curves under cyclic negative bias (c) and cyclic positive bias (d). (e) Time dependent current value shows the electric-field-driven dynamic resistance changes in NNO-NO. (f) Measurement delay time dependent I–V curves show the frequency dependent phenomenon.

To identify the electrical properties, current-bias curves are measured under different conditions. Figure 4a shows the schematic of the junctions with the gold top electrode and SRO bottom electrode. The length of gold square electrode is about 80 μm, large enough to cover both NO and NNO phases. For electrical testing, we applied a bias onto the top electrode, with the bottom electrode grounded for all measurements in this study. The initial current-bias curve of this setup exhibits both rectifying characteristic and memristive switching phenomenon in figure 4b. The input signal was provided in the inset, and the measurement delay (dwell time) is 1 second. This result reveals a possible working mode of an inverse-parallel connection of a rectifier and a memristor (upper inserted circuit in figure 4a). Previous work also shows that the Au-NNO/NO-SRO system forms an equivalent rectifier due to the summation of the Au/NO Schottky barrier and the NO/SRO n-n^+^ barrier^24^. So in the left negative bias of figure 4b, the current is largely restricted, while in the right positive bias part, the result exhibits a large hysteresis current trace. From 0 V to 10 V, the current increases sharply at about 8 V. This is the OFF state in memristor and reveals rectifier characteristic only. The vacancies dopants drift under the electric field through the most favorable diffusion paths, such as the phase boundaries, to form channels with a high electrical conductivity in the meantime. It represents a continuous current increasing even the bias decreases from 10 V to 8 V. And we attribute this abnormal current increasing to a possible discharge process in the thin film. Generally, NNO will display depolarization phenomenon when external electric field decreases gradually, and charge diffusion will lead to an extra current increase. Below 8 V, the current retrace does not go along the former trace showing rectifier characteristic (unlike what we just discussed when bias increases from 0 to 8 V), but shows a larger current value. This approximate linear trace shows that the device remains a constant resistance and the memristor keeps an ON state. Obviously, without a rectifier, the trace will be symmetrical in the first and third quadrants^11^,^26^, or the trace will change into another case if the device comprises a rectifier in concurrent paralleled with a memristor^1^,^27^.

## Discussion

**Figure 5:**
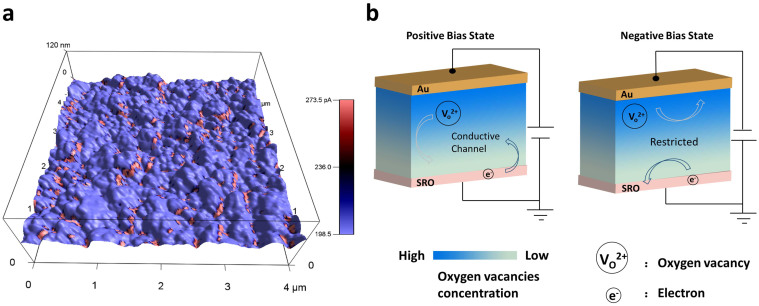
(a) Current mapping on topography of the NNO-NO thin film scanned by conductive AFM. (b) Illustration of positive charged oxygen vacancies migration and electron forming diverse conductive states under different external electrical fields.

A similar result is reported in TiO_2_ system, in which controlled oxygen vacancy plays a key role in the memristive switching^3^,^27^. Splayed current trace provides the ON and OFF state in TiO_2_ devices with controlled oxygen vacancy profiles. While in our case, we propose a general model to explain the switching behavior of the composite thin film. This model can be represented by an equivalent circuit (upper inset, figure 4a) which comprises a rectifier and a memristor in parallel back-to-back. Negative bias versus current (figure 4c) and positive bias versus current (figure 4d) are studied to present the details of dynamic resistance change under external electric field. In the first 5 traces under cyclic 0 V to −10 V to 0 V, the clockwise traces show the increasing resistance under the negative bias. Furthermore, in each cycle the current is smaller than the previous one, so the applied negative bias on the top electrode attracts the positively charged vacancies in the oxide. However, it behaves in an opposite way, when apply positive bias: the current increase shown by the anticlockwise trace reveals the decrease of resistance. A positive bias on the top electrode repels the vacancies from the conducting channel and attracts the electrons crossing the SRO and NO barrier^24^. Both two cases demonstrate the external electric field dependence of resistance shown in figure 5b.

Also, time dependence testing offers direct evidence about stability of current under positive or negative bias in figure 4e. The current decreases sharply in the beginning and then becomes stable gradually with slight increase under negative bias (green line), but it increases sharply under positive bias (red line). This difference can be understood as the inhomogenous oxygen vacancies concentrate in NO/NNO system, that top layer has a higher concentration of vacancies (evidence can be found in the I–V curve under alternative electric fields). The relative large mismatch in this self-assembly material also allows its vertical interfaces, the boundaries between two phases, to provide a large chance to form oxygen vacancy sites. These conductive channels can be found near the phase separation boundary clearly in figure 5a which shows the conductivity mapping of NNO-NO thin films scanned by conductive atomic force microscopy. Topography shows that the surface of the thin film is overlaid with a series of colors. As the color scale shows, red color stands for the large current value, while blue color represents the small current value. The red is mainly located near the foot of the “hills” revealing that the edge of the grain has much smaller resistance. The boundary represents incongruous electrical property suggesting that inhomogeneous phase separation forms conductive channel at the grain boundaries. Memristive switching mechanism is sensitive to the frequency of applied bias. We change the measure delay time to alter the frequency in each measurement in figure 4f. The decreasing measurement delay time from 100 ms to 1 ms (equivalent to the increase of measurement frequency) leads to a higher current. Because longer measurement delay time allows more vacancies drift via ionic conductivity at the same time, then the current increases. Ferroelectric materials can also show tuned I–V switch behaviours by polarization switch under electric field, that maximum current value occurs at the moment of polarization switching during bias increasing^28^. While all these switching behaviors found in the niobium based binary and ternary oxides composite thin films support the combination of rectifier and memristor with a characteristic ionic conductivity, rather than switching behaviour from ferroelectric polarization which would be triggered faster comparably also without any frequency-dependent hysteresis. Oxygen vacancies at the NNO/NO interface play an important role. Compared to other defects like impurity atoms and interstitial atoms inside ferroelectric NNO, the concentration of oxygen vacancy near interfaces is much larger due to the evident mismatch between NO and NNO. Large amount of vertical boundaries across the thin film form the oxygen vacancy channels and enhance the conductance as well. This self-assembly composite based new memristor device will significantly extends circuit functionality and produce new applications.

Benefiting from the film deposition technology, we grow Nb_2_O_5_ and NaNbO_3_ together as a composite thin film with a vertical heteroepitaxial nanostructure. Phases distribution and growth mechanism are confirmed by AFM and STEM. There is 15° mismatch angle between NNO (001) and NO (002), which decreases the difference between lattices and forms a diagonal direction as a symmetrical axis. Asymmetric hysteresis I–V curves demonstrate the memristive switching behavior of this NNO-NO thin film. An equivalent circuit combining a rectifier and a memristor in parallel helps to understand this switching process. Boundaries between NNO and NO phases are considered as the channel for oxygen vacancy that provides the possibility to change resistance dramatically. We believe that this finding may inspire research into developing new structure-tuned memristor devices.

## Methods

NaNbO_3_-Nb_2_O_5_ composite thin film with a composition ratio of 66 at.% NNO-34 at.% NO was deposited on (001) oriented LaAlO_3_ (LAO) substrates with SrRuO_3_ (SRO) buffer layer (as a bottom electrode) by pulsed laser deposition. This SRO layer with a thickness of 140 nm was firstly deposited at 750°C. NNO-NO target with stoichiometric ratio was synthesized by the traditional solid phase sintering. The spot size of the laser was about 2 mm^2^, which was focused on the surface of the target with energy density of 2 J*cm^−2^. The distance between the substrate and the target was 6 cm, and the base vacuum of the chamber was 10^−6^ Torr, while the oxygen pressure was 75 mTorr. Conductive Atomic force microscopy (C-AFM) images and piezoresponse force microscopy (PFM) images were taken by an atomic force microscopy (AFM, Cypher, Asylum Research) under the ORCA mode and Dual AC Resonance Tracking mode (DART)^29^, respectively. Scanning transmission electron microscopy (STEM) and energy-dispersive X-ray spectroscopy (EDS) analyses were acquired by probe spherical aberration corrected JEOL- ARM200F. A FEI Helios 600i Focused ion beam (FIB) was used to lift-out and prepare STEM samples. Gold square patterns with side length of 80 μm, acting as top electrodes, were deposited by sputtering using a metallic mask. We localized the conductive tip on the top of gold electrode, and then measured I–V curves by Agilent B2901A precision source/measure unit and Agilent 4155C semiconductor parameter analyzer. Measure delay time, as a setup parameter in measure unit, is equal to dwell time in the testing process.

## Author Contributions

L.L.L. and Y.D.Y. conceived the experiments. L.L.L., L.L. and Z.W. carried out experiments. L.L.L. wrote the manuscript. Y.L., Y.G.Y., D.Z., G.Y., J.Y. and D.V. discussed the data and the results, and commented the manuscript.
